# Scarlet Fever

**Published:** 1876-11

**Authors:** Lunsford P. Yandell

**Affiliations:** Louisville; Professor of Therapeutics and Clinical Medicine, University of Louisville


### SCARLET FEVER.

By LUNSFORD P. YANDELL, JR., M.D.,
Professor of Therapeutics and Clinical Medicine, University of Louisville.

This disease has existed to a serious extent in Louisville for some months, and although at no time really epidemic, yet it has been sufficiently abundant to make parents anxious, and to attract to it the profession's attention. It is a mortifying acknowledgment to make, but it is a fact that we have been acquainted with the disease for more than two centuries, and yet the profession is by no means of one mind either as to its natural history or cure. Hence I venture to publish my individual opinions on this open and vexed subject without fear of being charged with arrogance or dogmatism.

The contagiousness of scarlatina has been denied by not a few respectable physicians, but the same is true of variola, and there are medical men who even oppose vaccination. To my mind nothing is clearer than the contagiousness of scarlatina, although certainly less so than variola. As to the assertion that there can be no degrees of contagion any more than shades of virtue, it is unworthy of argument. On one point, however, we should all agree: any disease suspected of contagiousness should be treated as though indisputably so.

Being possessed unfortunately of no specific remedy for this specific disease, we should treat scarlatina without regard to its name, simply devoting our efforts to the control of symptoms and conditions.

Heat is probably the paramount source of danger in scarlatina, and it is the symptom over which we have most power. For its measurement there is but one reliable means, the thermometer; and for its reduction but one agent of indisputable potency, water. The form of aqueous application must be determined by circumstances, and the same is true of its temperature and duration. The patient's comfort must be considered, and a struggle avoided if possible. A protracted and moderately cool bath is better than one brief and decidedly cold. When practicable use the tub with tepid water, and gradually reduce its temperature to 70° or 60°, always avoiding an unpleasant degree of cold, and keeping the patient in half an hour, an hour, or even longer. Indeed there is no danger of too long a bath, if the patient enjoys it, and the heat of the body be not abnormally depressed.

Second to the tub-bath is the wet sheet. What has been said of the former applies to the latter. Properly applied, the water-treatment is without danger in scarlatina, as it is in typhoid and malarial fevers, variola, and other febrile affections. The terrible *bete noir* of scarlatina, dread of "taking cold," is an ancient error of apparently indestructible vitality, and one that does incalculable harm. To it we owe the close, hot, and foul sick-rooms so often encountered, and which so greatly enhance the mortality of scarlatina. Of course draughts and cold rooms are to be avoided. Anointing has some feeble power to reduce temperature, but its true office is in the stage of desquamation, where by allaying itching it promotes comfort.

Tincture of the muriate of iron is a valuable remedy in this as in all the blood-poisons. The tincture of the citro-muriate is equally efficacious and much more palatable. Emetics and nauseants are only justifiable when deficiency of secretion in mouth and throat exists, and purgatives may do good when the kidneys fail in their function. Arterial sedatives are of doubtful efficacy. Alcohol is applicable in the same conditions in this as in other diseases, and this and no more can be justly claimed for quinine. Antizymotics, though theoretically useful, are practically inert.

As for the local manifestations of scarlatina—whether in the throat, eyes, ears, nose, or elsewhere—there is no peculiar treatment. Cauterization and other violent remedies are to be avoided. Diarrhœa should not be checked unless it indisputably is doing harm. Deficiency of urine is best treated by cold water, externally and internally, and by digitalis. Scarlatinal albuminuria and dropsy incline to get well of themselves, and the credit awarded gallic acid in their removal is undeserved. Fresh air, cleanliness, and nourishment are of prime importance. Whatever form of food the stomach will accept should be allowed—whether it be fruit, flesh, milk, or bread—and cold drinking water and ice should be given *ad libitum*. There are no prophylactics to scarlet fever. Belladonna internally, and carbolic acid, camphor, and asafetida externally, are alone useful as *placebos* to parents and children old enough to know fear.

Trusting more to nature, and confiding less in drugs than physicians of a former day, we get better results from our practice than did our predecessors. The majority of cases recover under any treatment. Certain cases prove fatal in defiance of all curative measures. In most cases we have power to mitigate the severity of symptoms, enhance comfort, and assist nature to return to health.—*Louisville Medical News.*

